# Non-invasive assessment of mercury exposure in rodents and marsupials from a historically contaminated area of the Madeira River, Western Amazon

**DOI:** 10.1007/s00128-026-04260-9

**Published:** 2026-05-23

**Authors:** João Bezerra Facundo, Cássio da Silva Cabral, Mariluce Resende Messias, Wanderley Rodrigues Bastos, Wilson Gomez Manrique

**Affiliations:** 1 Postgraduate Program in Conservation and Use of Natural Resources – PPGReN, Federal University of Rondônia, Porto Velho, Rondônia Brazil; 2 Laboratory of Mammalogy and Terrestrial Vertebrates, Federal University of Rondônia, Porto Velho, Rondônia Brazil; 3 Wolfgang Christian Pfeiffer Environmental Biogeochemistry Laboratory, Federal University of Rondônia, Porto Velho, Rondônia Brazil; 4 Academic Department of Veterinary Medicine, Federal University of Rondônia, Rolim de Moura, Rondônia Brazil

**Keywords:** Arboreal mammal, Fur, Small mammals, Terrestrial mammals

## Abstract

The Lower Madeira River (Western Amazon) is a hotspot for mercury (Hg) contamination due to both natural geochemical enrichment and long-term anthropogenic activities. Total mercury (THg) concentrations were significantly higher in marsupials (median = 0.136 mg kg^−1^) than in rodents (median = 0.032 mg kg^−1^, *p* = 0.0006). Arboreal rodents showed lower Hg levels than omnivorous rodents. Terrestrial rodents showed higher Hg concentrations than arboreal rodents (*p* < 0.00010). Age did not influence Hg levels in the studied orders. The marsupial species *Marmosops* sp. (3.379 mg kg^−1^) and *Marmosa murina* (2.571 mg kg^−1^) showed the highest Hg concentrations, while in the rodent *Sphiggurus roosmalenorum* (n = 4), the concentrations remained below the detection limit. Atmospheric deposition is likely a source of Hg for herbivores, in addition to insects to supplement the diet. For omnivores, the main source of Hg is insects, which transport the element from the aquatic environment to the terrestrial environment.

## Introduction

The Lower Madeira River (Western Amazon) is a hotspot for (Hg) contamination due to geological and anthropogenic factors. The river’s high sediment load facilitates Hg transport (Bastos et al. 2020), which, upon deposition in floodplains, naturally enriches the environment. Furthermore, during the late twentieth century, the Madeira River was extensively exploited for gold mining, further increasing Hg levels (Pfeiffer and Lacerda 1988; Pestana et al. 2022). Other contributing anthropogenic pressures include deforestation and wildfires (De Lacerda et al. 2024), changes in land use and occupation (Monteiro et al. 2024), and the construction of hydroelectric reservoirs, which can enhance the environment’s methylation potential (Pestana et al. 2019).

Certain animal groups are highly sensitive to environmental shifts and are widely recognized as effective bioindicators, such as rodents and marsupials (Ballová et al. 2020; Oliveira and Del-Claro 2003). The order of rodents is the largest order of mammals, characterized by the continuous growth of their incisors. They are critical seed dispersers and natural controllers of invertebrates (Antunes et al. 2022). The order Didelphimorphia is composed of New World marsupials. A striking characteristic of this order is the presence of the marsupium, an external pouch where offspring complete their development. They are generalist and opportunistic animals, with nocturnal habits and the use of scent marking odor and thanatosis as defense mechanisms.

Despite the vast literature regarding Hg contamination in the Amazon, most studies focus on human exposure (Bastos et al. 2006; Mendes et al. 2021; Canela et al. 2024) and aquatic biota, primarily fish (Bastos et al. 2015; Mussy et al. 2022; Cabral et al. 2025), caimans, and river dolphins (Correia et al. 2014; Gomes et al. 2020; Barbosa et al. 2021). Consequently, significant knowledge gaps remain in the toxicology of small terrestrial mammals, such as rodents and marsupials, in areas historically impacted by mining and environmental modifications, such as hydroelectric plants (Maclntosh, 2019). While rodents are predominantly herbivores and marsupials are generally opportunistic omnivores, both groups are subject to ecological imbalance despite occupying different positions in most cases. To monitor Hg levels in these mammals, fur has been used as a non-invasive biomarker since it reflects evidence of exposure over a long period of time, and causes minimal stress to individuals, obviating the need for euthanasia (D’Havé et al. 2006; Lurz et al. 2017; Treu et al. 2018; Lopes et al. 2020).

Therefore, the objective of this study is to assess and compare THg concentrations in the fur of small mammals (rodents and marsupials) inhabiting an area historically influenced by and contaminated by Hg in the Madeira River. The study aims to verify whether trophic levels and foraging habitat of these small terrestrial mammals influence THg bioaccumulation, thereby providing novel data for these orders in the Madeira River region. Despite their ecological relevance and sensitivity to environmental contamination, small terrestrial mammals remain critically underrepresented in Hg biomonitoring studies within tropical regions.

## Materials and methods

### Study area and sampling

The study area encompasses the area of ​​influence of the Santo Antônio Hydroelectric Power Plant, a region known as the Lower Madeira River within the municipality of Porto Velho, Rondônia. Fur samples from the orders Rodentia and Didelphimorphia were obtained from the mammal collection of the Laboratory of Mammalogy and Terrestrial Vertebrates (LabMasto) at the Federal University of Rondônia using stainless steel scissors. The specimens selected for this study originated from fauna rescue conducted in the direct ​​influence area of the Santo Antônio HPP. In total 63 samples were collected (56 rodents and 7 marsupials). These specimens originated from fauna rescues carried out between 2008 and 2014. Data such as age (adults and juveniles/pups) were obtained from the LabMasto database, having been previously determined and reviewed by mammal specialists. Information on habitat and trophic guild was determined based on specialized literature (Table 1).

**Table 1:** Median levels of total mercury (mg/kg) and range in the fur of marsupials (Didelphimorphia) and rodents (Rodentia) from the area affected by the Santo Antônio Hydroelectric Plant, Lower Madeira River, Porto Velho, RO

Order/Species	n	Median	Range	Habit	Trophic guild	References
Didelphimorphia						
* Caluromys ianatus*	03	0.136	0.082–0.180	Arboreal	Frugivore/Omnivore	Emmons and Feer (1997)
* Marmosa murina*	01	2.571	–	Arboreal	Omnivore	Voss and Jansa (2009)
* Marmosops* sp.	01	3.379	–	Arboreal/Terrestrial	Insectivorous/Omnivore	Palma (1996)
* Metachirus nudicaudatus*	01	0.077	–	Terrestrial	Insectivorous	Astúa de Moraes (1998)
* Micoureus demerarae*	01	0.083	–	Arboreal	Omnivore	Charles-Dominique et al. (1981)
Rodentia						
* Coendou prehensilis*	10	0.005	< LOD–0.229	Arboreal	Frugivore/Granivore	Patton et al. (2015)
* Cuniculus paca*	08	0.020	0.008–0.051	Terrestrial	Frugivore	Patton et al. (2015)
* Dactylomys dactylinus*	02	0.006	0.003–0.009	Arboreal	Herbivore	Patton et al. (2015)
* Hydrochoerus hydrochaeris*	01	0.039	–	Semi-aquatic	Herbivore	Patton et al. (2015)
* Isothrix bistriata*	08	0.023	< LOD–0.073	Arboreal	Herbivore	Patton et al. (2015)
* Mesomys hispidus*	02	0.011	< LOD–0.023	Arboreal	Herbivore	Patton et al. (2015)
* Nectomys rattus*	01	0.317	0.317–1.001	Semi-aquatic	Omnivore	Santori et al. (2008)
* Oecomys* sp.	01	0.021	–	Arboreal	Frugivore/Granivore	Patton et al. (2015)
* Proechimys gardineri*	13	0.044	0.027–0.590	Terrestrial	Omnivore	Maldonado et al. (2019)
* Rattus rattus*	06	0.180	0.065–0.331	Terrestrial	Omnivore	Santori et al. (2008)
* Sphiggurus roosmalenorum*	04	< LOD	–	Arboreal	Frugivore	Patton et al. (2015)

Data on habits and trophic guilds were compiled from a review of the scientific literature (see “References” column). < LOD: value below the limit of detection

### Chemical preparation and determination of total mercury.

The samples were washed with 0.01% (w/v) ethylenediaminetetraacetic acid (EDTA) for twenty-four hours to remove adsorbed impurities. Subsequently, they were washed with ultrapure water (resistivity of 18.2 MΩ.cm^−1^; Milli-Q, Millipore, Bedford, MA, USA). After oven-drying, the samples were finely cut using acid-cleaned stainless-steel scissors and weighed on a previously calibrated analytical balance. Approximately 80 mg of each sample was weighed into 100 mL glass tubes. To each tube, 4.0 mL of an acidic mixture (1:1, HNO_3_:H_2_SO_4_; Merck^®^) was added and the samples were placed in a digestion block (TE-007MP, Tecnal, Piracicaba, Brazil) at 70 ºC for 30 min. After cooling, 5.0 mL of 55 (w/v) potassium permanganate (KMnO_4_) was added, and the samples remained in the digestion block for an additional 20 min. At room temperature, the samples were left overnight. The following day drops of 12% (w/v) hydroxylamine hydrochloride (HN_2_OH.HCl; Merck^®^) were added to remove excess KMnO_4_. The samples were transferred to volumetric flasks, and the final volume was adjusted to 10.0 mL with ultrapure water (Milli-Q, Millipore, Bedford, MA, USA). The THg concentration was determined Cold Vapor Atomic Absorption Spectrometry (CV-AAS) using an automated spectrometer (FIMS-400, PerkinElmer, Waltham, MA, US) (Bastos et al. 1998).

### Analytical quality control

To ensure analytical quality and data reliability, reagent blanks were analyzed to verify purity, and certified reference material (CRM) DORM-2 (National Research Council Canada 1999) was utilized. All samples, including the CRM, were analyzed in duplicate. The recovery rate (113.5%) is consistent with previously reported values for hair matrices and reflects matrix-specific analytical variability** (**D’Havé et al. 2006). The limit of detection (LOD) was 0.0001 mg kg^−1^, and the limit of quantification (LOQ) was 0.001 mg kg^−1^. The LOD and LOQ were calculated using the expressions LOD = 3.3(σ/S) and LOQ = 10 (σ/S), where σ is the standard deviation of the blank measurements of the calibration curve and S is the slope of the calibration curve. All material used in the preparation and analysis of the samples was previously decontaminated with a nitric acid bath (3%, Merck^®^).

### Data analysis

Data normality was assessed using the Shapiro–Wilk test. Due to the non-normal distribution, the Mann–Whitney test was used for comparisons between orders, trophic guilds, age groups (adults and juveniles/young), and habits. The Kruskal–Wallis test and Dunn’s post hoc test were used for comparisons between different collection years. Concentrations below the limit of detection (LOD) were not included in the statistical analyses (n = 7). The significance level was set at < 0.05 for all tests, using GraphPad Prism 8.0.1 software.

## Results

**Fig. 1:**
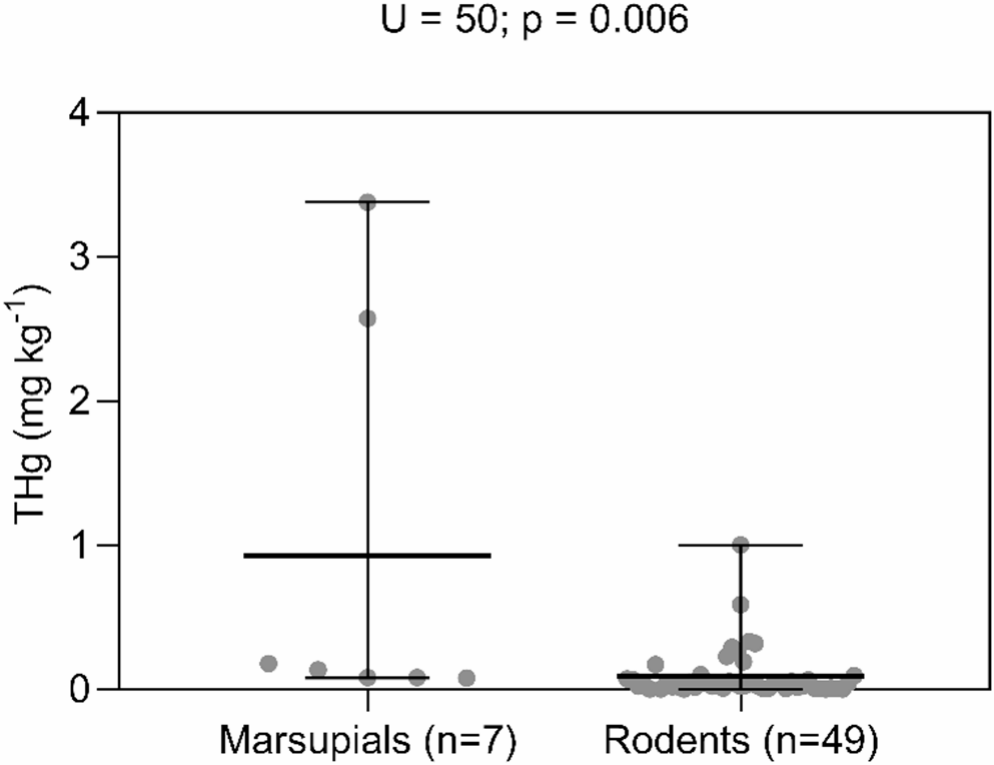
Total mercury (THg) concentrations in hair samples from marsupials (n = 7) and rodents (n = 49) collected in the area surrounding the Santo Antônio Hydroelectric Plant, Lower Madeira River, Porto Velho, RO. The horizontal bars represent the median and the interquartile range

**Fig. 2:**
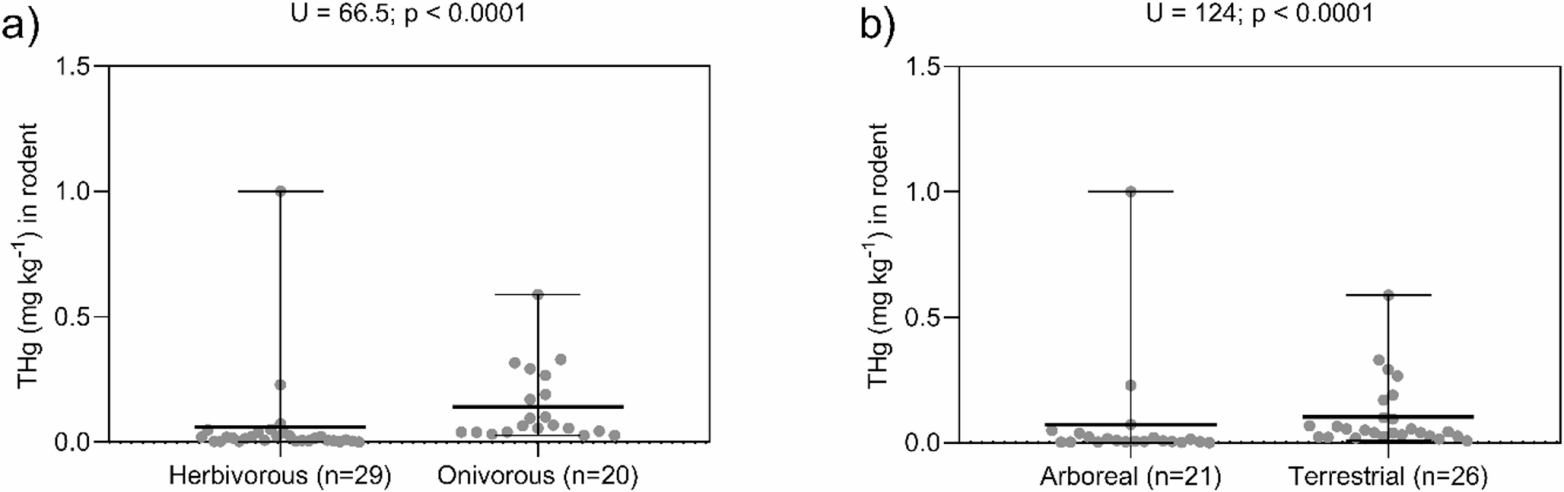
Influence of diet and habitat on THg bioaccumulation in hair samples from rodents collected in the area around the Santo Antônio Hydroelectric Power Plant, Lower Madeira River, Porto Velho, RO. **a** Difference between herbivorous and omnivorous rodents, where THg levels are higher in herbivorous rodents (*p* < 0.0001) and **b** shows that THg concentrations are higher in terrestrial rodents than in arboreal rodents (*p* < 0.0001)

THg levels were determined for 63 individuals. The median THg concentration was significantly higher in the order Didelphimorphia (0.136 mg kg^−1^) than in Rodentia (0.032 mg kg^−1^). Notably, one individual from the order Didelphimorphia exhibited a peak concentration of 3.379 mg kg^−1^, while THg levels in seven rodent specimens were below the LOD. The Mann–Whitney test indicated a significant difference between the two orders (U = 50; *p* = 0.0006) (Fig. 1) Regarding age classes within Rodentia, no significant difference (*p* = 0.0838) in THg concentrations between adults (0.038 mg kg^**−1**^; n = 40) and juveniles (0.019 mg kg^**−1**^; n = 9). The Kruskal–Wallis test indicated that within the order Rodentia, THg concentrations were significantly higher in animals rescued in 2008 compared to 2010 (KW = 17.30; *p* = 0.004). Furthermore, significant differences were found between trophic guilds, with omnivorous rodents exhibiting higher THg levels than predominantly herbivorous ones (U = 66.5; *p* < 0.0001) (Fig. 2a). Finally, foraging habitat also influenced bioaccumulation, as THg was higher in terrestrial rodents than in arboreal species (U = 124; *p* < 0.0001) (Fig. 2b).

## Discussion

The statistical difference (*p* = 0.0006) between THg concentrations in rodents (0.032 mg kg^−1^) and marsupials (0.136 mg kg^−1^) (Fig. 1) highlights the distinction between taxa with respect to their trophic niche. The trophic level among rodent species supported this pattern, given that omnivorous rodents showed higher THg concentrations than herbivorous rodents (U = 66.5; *p* < 0.0001) (Fig. 2a). Thus, it was observed that diet is the main factor in Hg bioaccumulation in this location, historically impacted by anthropogenic actions of Hg contamination. Our data showed a peak concentration of 3.379 mg kg^−1^ of THg in *Marmosops* sp. (Table 1), a marsupial with habits ranging from insectivorous to omnivorous, with a diet rich in invertebrates (Malaquias 2018), demonstrating that prey such as invertebrates are important transporters of organic Hg from the organic environment to the terrestrial environment (Moyo 2020; Bandouchova et al. 2024).

Insects participate in the Hg cycle within both terrestrial and aquatic environments. In terrestrial ecosystems, insects bioaccumulate Hg from the soil and air and actively contribute to the Hg cycle as both predators and prey, as well as through the decomposition of organic matter (Rebolloso-Hernandez et al. 2023). Notably, Moyo (2020) estimated that insects annually transfer approximately 650 kg of MeHg from aquatic to terrestrial environments, originating from both rivers and lakes. In cicadas, Zheng et al. (2010) observed average Hg concentrations of 2.640 mg kg^−1^ in the body and 0.500 mg kg^−1^ in the exuviae, demonstrating that cicadas are significant sources of Hg and that substantial concentrations accumulate in the exoskeleton. Furthermore, the exoskeleton can serve indirectly as a pathway for Hg elimination through molting (Zheng et al. 2010). In insects, organic Hg exhibits a greater capacity for absorption and bioaccumulated than inorganic Hg (Rebolloso-Hernández et al. 2023), leading to higher rate of bioaccumulation rates for insect predators. In a region historically affected by Hg contamination, with concerned levels of Hg in fish and among riverside communities (Mendes et al. 2021; Mussy et al. 2022; Canela et al. 2024; Cabral et al. 2025), insects may serve as a source of aquatic Hg transfer to terrestrial animals, resulting in high levels as observed in *Marmosa murina* and *Marmosops* sp. (Table 1) (Moyo 2020).

Another important factor in the exposure and bioaccumulation of Hg in rodents is foraging habitat (Fig. 2b). Terrestrial rodents have higher concentrations of THg than arboreal rodents (U = 124; *p* < 0.0001). Soil and leaf litter are natural reservoirs of Hg in the Amazon, being one of the sources of Hg for animals with terrestrial habitats (Lechler 2000; Fadini and Jardim 2001). In these compartments, microhabitats, the abundance of organic matter, and humidity present favorable conditions for microbial activity, transforming inorganic Hg into organic Hg, which is more toxic to animal health (Yang Guang et al. 2019; Ma et al. 2022). This methylation process of Hg makes it more bioavailable to living organisms, such as *P. garderi*, which use the substrate for foraging (Maldonado et al. 2019).

In contrast, our data showed that most arboreal rodents are less exposed to Hg (Fig. 2b). Since roots act as a natural barrier against Hg translocation in plants (Beauford et al. 1977), the exposure of these herbivorous and frugivorous animals is mainly occurring through atmospheric deposition on leaves via stomata (Mallongi et al. 2014; Zhou et al. 2021; Gerson et al. 2022; Gustin et al. 2022). In general, Hg ingested via vegetation is in its inorganic form, with a low absorption rate (7 to 15%) and a higher excretion rate compared to methylmercury (Park and Zheng 2012), which explains the low concentrations found in arboreal rodents and the absence of significant differences between adults and juveniles/pups (*p *= 0.0838).

The study area, under the influence of the Santo Antônio and Jirau hydroelectric dams, is situated within a context of intense environmental changes that enhance Hg methylation in aquatic biota (Hylander et al. 2006; Hsu-Kim et al. 2018; Pestana et al. 2019; Poulin et al. 2023). In addition, the history of artisanal gold mining in the Madeira River basin released large quantities of metallic Hg, which, upon volatilization, is deposited in the adjacent vegetation (Malm 1998; Gerson et al. 2022). This complex interaction of anthropogenic and natural sources creates a scenario where terrestrial fauna is continuously exposed to the metal deposited on leaves and accumulated in the soil, exacerbating the risks of biomagnification in local food webs.

In summary, our dataset indicates that Hg bioaccumulation in marsupials and rodents in the area influenced by the Santos Antônio Hydroelectric Power Plant is driven by an interaction between foraging habits and trophic guild (Fig. 2). While arboreal herbivores have low exposure via a diet of leaves and fruits, terrestrial omnivores are more vulnerable to bioaccumulation through a diet of prey that transfer Hg via the food chain, such as insects.

## Conclusion

The results confirm the effectiveness of fur as a biomarker for monitoring THg in rodents and marsupials. Total mercury levels are significantly influenced by feeding habits, being higher in marsupials. For rodents, two factors affect Hg concentrations: trophic level and foraging habitat. Even within the same order, diet is determinant: omnivorous rodents are more exposed than herbivores. Additionally, foraging habitat proved to be a critical factor, with terrestrial rodents showing greater bioaccumulation of THg than arboreal species.

However, certain limitations, such as the absence of complementary environmental data (soil, water, and vegetation), the small sample size for some species, and the lack of information on the chemical speciation of accumulated Hg, prevent more precise toxicological interpretations. These findings highlight the importance of incorporating small terrestrial mammals into Hg monitoring programs in tropical ecosystems affected by long-term anthropogenic disturbances.

## Data Availability

The datasets generated and/or analyzed during the current study are available from the corresponding author.
